# Diagnostic and Prognostic Value of Soluble Urokinase-type Plasminogen Activator Receptor (suPAR) in Focal Segmental Glomerulosclerosis and Impact of Detection Method

**DOI:** 10.1038/s41598-019-50405-8

**Published:** 2019-09-24

**Authors:** Wolfgang Winnicki, Gere Sunder-Plassmann, Gürkan Sengölge, Ammon Handisurya, Harald Herkner, Christoph Kornauth, Bernhard Bielesz, Ludwig Wagner, Željko Kikić, Sahra Pajenda, Thomas Reiter, Benjamin Schairer, Alice Schmidt

**Affiliations:** 1 Department of Medicine III, Division of Nephrology and Dialysis, Medical University of Vienna, Vienna, Austria; 2 Department of Emergency Medicine, Medical University of Vienna, Vienna, Austria; 3 Clinical Institute of Pathology, Medical University of Vienna, Vienna, Austria

**Keywords:** Focal segmental glomerulosclerosis, Glomerular diseases

## Abstract

The plasma soluble urokinase-type plasminogen activator receptor (suPAR) is a biomarker for focal segmental glomerulosclerosis (FSGS), but its value is under discussion because of ambiguous results arising from different ELISA methods in previous studies. The aim of this study was to compare diagnostic performance of two leading suPAR ELISA kits and examine four objectives in 146 subjects: (1) plasma suPAR levels according to glomerular disease (primary, secondary and recurrent FSGS after kidney transplantation, other glomerulonephritis) and in healthy controls; (2) suPAR levels based on glomerular filtration rate; (3) sensitivity and specificity of suPAR for FSGS diagnosis and determination of optimal cut-offs; (4) suPAR as prognostic tool. Patients with FSGS showed significant higher suPAR values than patients with other glomerulonephritis and healthy individuals. This applied to subjects with and without chronic kidney disease. Although both suPARnostic™ assay and Quantikine Human uPAR ELISA Kit exerted high sensitivity and specificity for FSGS diagnosis, their cut-off values of 4.644 ng/mL and 2.789 ng/mL were significantly different. Higher suPAR was furthermore predictive for progression to end-stage renal disease. In summary, suPAR values must be interpreted in the context of population and test methods used. Knowing test specific cut-offs makes suPAR a valuable biomarker for FSGS.

## Introduction

Focal segmental glomerulosclerosis (FSGS) represents up to 20 percent of glomerular disease and is a leading cause of end-stage renal failure^1^. It affects native kidneys as well as renal allografts and the rate of recurrence of disease after transplantation is close to 30% in FSGS patients^2^.

The pathophysiology and etiology of FSGS is still unclear^3^. However, it has been speculated that certain circulating factors might contribute to the initiation of renal injury^4^ as evidenced by recurrence of FSGS after kidney transplantation^5^ and the induction of remission after plasma exchange in subjects with recurrent FSGS early post renal transplantation^6^. Further evidence of potential circulating factors is the induction of proteinuria in kidneys of rats by administration of serum from FSGS patients^7^ and the transmission of FSGS from mother to child^8^. In addition, it was reported that re-transplantation of a renal allograft with rapidly recurrent FSGS in a recipient with primary FSGS as underlying disease into a second recipient without native FSGS resulted in complete remission^9,10^.

Several proteins have been suggested as the causative circulating factors responsible for the occurrence of primary FSGS or recurrence of disease after kidney transplantation, including cardiotrophin-like cytokine-1 (CLC-1)^11^, vasodilator-stimulated phosphoprotein (VASP)^12^, anti-CD40 antibodies^13^ and soluble urokinase-type plasminogen activator receptor (suPAR)^14^. The most investigated factor in this context to date is suPAR^15^.

The soluble urokinase-type plasminogen activator receptor (suPAR) is derived from the urokinase-type plasminogen activator receptor (uPAR), a membrane bound receptor expressed at the surface of multiple cell types such as immune cells and vascular endothelial cells^16^. By cleavage of its glyxosylphospatidylinositol anchor, uPAR can be released from the plasma membrane as soluble multi-domain signaling molecule (suPAR)^5^. In proteinuric kidney diseases, in particular FSGS, uPAR expression in the kidney and suPAR concentrations in plasma are elevated. As a result of high suPAR and consequential podocyte β3 integrin activation, it effects podocyte foot process effacement and disruption of the glomerular barrier leading to proteinuria^16^.

The suPAR levels can be detected in plasma, serum, urine and other body fluids^17^ and are stable in healthy individuals. However, increased plasma levels can be detected not only in FSGS patients, but also under various disease conditions, such as sepsis^18^, liver cirrhosis^19^, immunological disorders^20^ and malignancies^21^ suggesting a role of suPAR as acute phase protein^17^.

The impact of circulating suPAR as causative factor for FSGS is yet under debate. In initial studies, using a proposed suPAR cut-off level of 3.0 ng/mL, increased suPAR levels were identified in up to two-thirds of patients with FSGS^22^, which was significantly higher compared to patients with other forms of glomerular diseases^14^. Furthermore, high suPAR levels were reported to be predictive for recurrence of FSGS after kidney transplantation^14^. It has therefore been suggested that suPAR may represent the circulating factor causing FSGS^22^ and, as a biomarker, may support the often sophisticated FSGS diagnosis.

The diagnosis of FSGS is based on histopathologic findings in representative kidney biopsies and accordingly analysis of at least 15 serial sections is recommended^23^. The quality of a renal biopsy depends on the number of glomeruli and 10–15 glomeruli are considered optimal^24^. The injury of podocytes initiates the disease process of FSGS, leading to the classical focal distribution of sclerosis with a segmental pattern within the glomeruli^25^. However, due to the focal and segmental characteristics of FSGS, histopathological features may be missed and depend on biopsy site and time of biopsy. Biopsy results do not always reflect clinical manifestations^3,26^, therefore, additional tests may facilitate diagnosis of FSGS^3^.

The usefulness of suPAR as a biomarker is promising, but still a matter of debate due to ambiguous results in previous studies which have shown that suPAR levels depended on the degree of renal insufficiency, did not correlate with the extent of proteinuria and were also increased in other kidney diseases than FSGS^27–30^. Furthermore, the role of suPAR in progression of FSGS and chronic kidney disease is discussed in the scientific community^1,31,32^. Varying results in former investigations might partly arise from application of different ELISA kits using differing reagents and antibodies. The aim of our study was therefore to compare the diagnostic and prognostic performance of two leading suPAR ELISA kits that use different capture and detection antibodies of non-identical hosts. We have determined four outcomes using both Elisa kits side-by-side and analyzed their results:

(1) plasma suPAR levels according to glomerular disease (primary FSGS, secondary FSGS, recurrence of FSGS after kidney transplantation, other glomerulonephritis (GN) and in healthy controls; (2) plasma suPAR levels according to kidney function (eGFR); (3) sensitivity and specificity of suPAR levels for FSGS diagnosis after determination of an optimal cut-off value and (4) assessment of suPAR as prognostic marker.

## Methods

### Study design

This retrospective cross-sectional study was conducted to compare plasma levels of suPAR in patients with FSGS, other forms of glomerulonephritis and healthy individuals. We further compared suPAR levels in patients with primary FSGS, secondary FSGS and recurrence of FSGS after kidney transplantation.

### Study population and recruitment

We measured plasma suPAR in 146 adults over 18 years. Overall 27 patients with primary FSGS, 21 subjects with secondary FSGS and 6 patients with recurrence of FSGS after renal transplantation with clinical and histologic data recorded at the Medical University of Vienna were included in this study. The primary sample collection was carried out between 06/2013 and 04/2015 and follow-ups were performed until 12/2018. Clinical and histological data were obtained at the date of presentation. Moreover, 60 patients with other forms of GN and 32 healthy individuals were used as age- and gender-matched controls (Table 1). Patients with “Other GN” included subjects with membranous GN, minimal change GN, immune complex GN, anti-GBM GN, IgA GN, Lupus GN, interstitial GN and patients with unspecified GN as given in Supplementary Table S1.

**Table 1:** Baseline Characteristics and plasma suPAR levels of Study Patients.

	FSGS total	Primary FSGS	Secondary FSGS	Recurrence of FSGS in RTX	Other GN	Healthy controls	P-value°
Number of subjects	54	27	21	6	60	32	
**Characteristics**							
- Age (years)	45.1 ± 14.7	48.4 ± 13.0	45.1 ± 15.4	30.2 ± 11.1	47.4 ± 16.6	42.3 ± 8.3	0.44
- Gender - male number (%)	33 (61)	16 (59)	15 (71)	2 (33)	32 (53)	16 (50)	0.57
- White - number (%)	48 (89)	25 (93)	17 (81)	6 (100)	57 (95)	30 (94)	0.19
- MAP (mmHG)	97 ± 9	98 ± 6	103 ± 6	88 ± 16	90 ± 11	92 ± 6	0.14
- Treated with RAS blockade - number (%)	44 (81)	22 (81)	17 (81)	5 (83)	43 (72)	0 (0)	0.42
- Treated with immunosuppression - number (%)	34 (63)	21 (78)	7 (33)	6 (100)	29 (48)	0 (0)	0.06
**Coexisting or prior illness – number (%)**							
- Arterial hypertension - number (%)	48 (89)	24 (89)	18 (85)	6 (100)	48 (80)	0 (0)	0.35
- Diabetes mellitus - number (%)	12 (22)	6 (22)	5 (24)	1 (17)	6 (10)	0 (0)	0.16
**Laboratory parameters**							
- Serum albumin (g/dL)	37.47 ± 7.27	35.81 ± 8.52	40.39 ± 3.69	35.23 ± 8.13	36.47 ± 7.61	ns	0.50
- Serum total cholesterol (mg/dL)	218.40 ± 62.08	230.24 ± 70.39	208.63 ± 55.89	200.00 ± 35.60	234.65 ± 67.46	ns	0.28
- Serum C-reactive protein (mg/dL)	0.41 ± 0.47	0.38 ± 0.56	0.48 ± 0.41	0.27 ± 0.20	0.23 ± 0.25	0.09 ± 0.08	0.02
- Serum creatinine (mg/dL)	2.19 ± 1.89	1.80 ± 1.22	2.31 ± 2.01	3.51 ± 3.30	1.40 ± 1.35	0.86 ± 0.11	0.0004
- eGFR (mL/min/1.73 m^2^)	52.37 ± 31.61	55.90 ± 32.13	51.61 ± 32.04	39.12 ± 28.87	75.85 ± 33.48	99.95 ± 15.89	0.0003
- Microscopic hematuria, n (%)	8 (15)	3 (11)	3 (14)	2 (33)	12 (20)	ns	0.47
- Urine albumin/creatinine ratio (mg/g)	1582 ± 1728	1627 ± 1774	1337 ± 1531	2376 ± 2272	1813 ± 2137	ns	0.89
- Urine protein/creatinine ratio (mg/g)	2316 ± 2325	2435 ± 2331	1848 ± 2101	3422 ± 2986	2526 ± 2913	ns	0.85
**Plasma suPAR levels (ng/mL)**							
SuPAR (suPARnostic™ assay)*	6.58 ± 2.25	6.65 ± 2.26	6.40 ± 2.46	6.96 ± 1.61	3.28 ± 1.47	1.53 ± 0.60	0.0001
SuPAR (Quantikine Human uPAR)**	3.54 ± 1.03	3.60 ± 1.14	3.44 ± 0.85	3.65 ± 1.26	3.07 ± 1.06	2.07 ± 0.50	0.0026

Plus-minus values are means ± standard deviation. Numbers in brackets indicate percentage.

Abbreviations: eGFR, estimated glomerular filtration rate; FSGS, focal segmental glomerulosclerosis; GN, glomerulonephritis; MAP, mean arterial pressure; ns, not studied; RAS, renin angiotensin system; RTX, renal transplantation.

°P-value of FSGS total vs. Other GN.

*Plasma suPAR value (ng/mL) measured by suPARnostic™ assay.

**Plasma suPAR value (ng/mL) measured by Quantikine Human uPAR ELISA Kit.

Exclusion criteria were age <18 years, active inflammatory disease, chronic liver disease, malignancy, pregnancy and any illicit drug use. Patients with FSGS or other GN whose renal biopsy specimen contained less than 10 glomeruli were also excluded.

We first examined the association between baseline suPAR levels and measures of kidney function (eGFR, urine protein/creatinine ratio and albumin/creatinine ratio) in all study subjects. We also investigated the association of suPAR and change in eGFR and further clinical parameters and outcomes during follow-up in all 54 FSGS patients during a follow-up period of 5 years.

### Histopathology

The kidney biopsy was performed at the time of diagnosis. Biopsy specimens were formalin-fixed, paraffin embedded, cut at 2 µm and histologically evaluated by light microscopy, indirect immunohistochemistry and electron microscopy following institutional guidelines. All of the biopsy slides were reviewed by two pathologists blinded to clinical data of the patients.

### Sample collection

Fasting venous blood samples were collected of 54 FSGS patients and samples from 92 age- and gender matched patients with other forms of GN or healthy individuals as normal controls. Plasma was separated within 30 minutes following blood draw and stored in multiple aliquots at −80 °C until analyzed. Repeated freezing and thawing cycles of the samples were avoided.

### Plasma suPAR measurement

Concentration of plasma suPAR in human subjects were detected using the suPARnostic™ assay (Virogates, Copenhagen, Denmark) and the Quantikine Human uPAR Immunoassay (R&D Systems, Minneapolis, MN, USA)^1^, following the manufacturers protocol. Both kits are measuring suPAR concentrations and minimum detectable levels are 100 pg/mL and 33 pg/mL, respectively. A schematic illustration of both kits is shown as Supplementary Fig. S1.

#### suPARnostic™ assay (Virogates, Copenhagen, Denmark)

The suPARnostic kit (ViroGates A/S, Birkerød, Denmark) represents a double monoclonal antibody sandwich assay that measures total suPAR, including both full-length and cleaved forms. Plates are coated with catching rat monoclonal antibody. Horseradish peroxidase (HRP)-labeled detection mouse monoclonal antibody is mixed in a volume of 225 μL of Peroxidase Conjugate solution together with 25 μL of plasma. Out of this, 100 μL was transferred (in duplicates) to the pre-coated immunoassay plate and incubated for one hour. After washing procedures, 100 μL of the TMB substrate (3,3′,5,5′ tetramethylbenzidine) was added to each well. The substrate/chromogen reaction was stopped after 10 minutes by the Stop Solution (0.45 M sulphuric acid - H_2_SO_4)_. All incubations were performed at room temperature in the dark.

Plasma sample concentrations were calculate according to the standard curve included in each plate. The intra‐assay variation of the kit is given as 2.8% and the inter-assay variation as 9.2%^33^. Samples were randomly distributed between kits of five different lot numbers and were all measured in duplicates. All samples had duplicate coefficients of variants (CVs) <10%.

#### Quantikine Human uPAR Immunoassay (R&D Systems, Minneapolis, MN, USA)

With regard to the Quantikine Human uPAR Immunoassay, 100 µL assay diluent was applied to every well of 96-well polystyrene microplates that were precoated with a mouse monoclonal antibody, followed by 50 µL plasma and incubated for 2 hours at ambient temperature. After incubation and washing procedure, horseradish peroxidase–conjugated polyclonal antibodies against uPAR were admixed and incubated for 2 hours at ambient temperature. After repeated washing, a substrate solution was applied to every well and incubated for further 30 minutes at ambient temperature while protecting it from light. Finally, 50 µL of stop solution was admixed in every well and the absorbance was detected at 450 nm over 30 minutes using an ELISA reader^1,34^. The intra‐assay variation of the kit is given as 7.5% and the inter-assay variation as 5.9%. Samples were randomly distributed between kits of five different lot numbers and were all measured in duplicates. All samples had duplicate coefficients of variants (CVs) <10%.

### Study procedures

#### Laboratory diagnostic for routine parameters

All laboratory parameters were determined at the Department of Laboratory Medicine, Medical University of Vienna, which has a certified (ISO 9001:2008) and accredited (ISO 15189:2008) quality management system. The eGFR was calculated using the chronic kidney disease EPI equation^35^.

#### Outcomes

The current study investigated the association of FSGS status and suPAR levels in plasma measured by different ELISA kits. Furthermore, a prognostic assessment of suPAR was performed for eGFR dynamics, ESRD and death.

### Statistical analyses

Categorized data are presented as absolute counts and relative frequencies (percent). We presented continuous data as means ± standard deviation (SD). To compare baseline variables between study groups we used the Mann-Whitney U test, the median test or the Fisher’s exact test, as appropriate. To compare difference in suPAR levels from different test kits we used the t-test with H_0_: delta = 0, calculating disease group specific estimates with respective 95% confidence intervals.

We calculated standard metrics of diagnostic test accuracy including sensitivity and specificity of suPAR levels (index tests) for identifying patients with FSGS (reference standard) independently. We estimated the optimal cut-off by maximizing the sensitivity-specificity product according to Liu’s method^36^ using the adjustment method suggested by Fluss *et al*.^37^. The confidence interval for the cut-off value was estimated by bootstrapping.

For prognostic assessment of suPAR (main co-variable) for eGFR dynamics, ESRD, and death (individual outcomes) multivariable regression analyses were employed. For eGFR dynamics linear regression, for ESRD and death logistic regression were used. Co-variables were sex (male vs female), age (years), eGFR (mL/min) and initial urine protein/creatinine ratio (mg/g). We report the estimates from the regression analysis with a 95% confidence interval and the corresponding P-values from the Wald test. We furthermore conducted a sensitivity analysis in which differences in eGFR, urine protein/creatinine ratio and albumin/creatinine ratio values were set to zero after loss of renal function.

Statistical analysis and data management was carried out with Stata 14 for Mac (Stata Corp, College Station, TX, USA), GraphPad Prism (© GraphPad Prism version 7.00 for Windows, GraphPad Software, La Jolla California, USA) and MS Excel (© Microsoft, Redmont, WA).

All tests conducted were two-sided and a P-value below 0.05 was considered significant.

## Results

### Study population

This study analyzed demographic data, laboratory parameters and plasma suPAR levels of 54 patients with FSGS (27 patients with primary FSGS, 21 with secondary FSGS and 6 with recurrence of FSGS in the renal allograft) and 92 controls (60 patients with glomerulonephritis other than FSGS termed “Other GN” and 32 healthy individuals) as measured by two leading ELISA assays. Demographic and clinical data of the entire study cohort are given in Table 1.

### Plasma suPAR level according to glomerular disease

**Figure 1:**
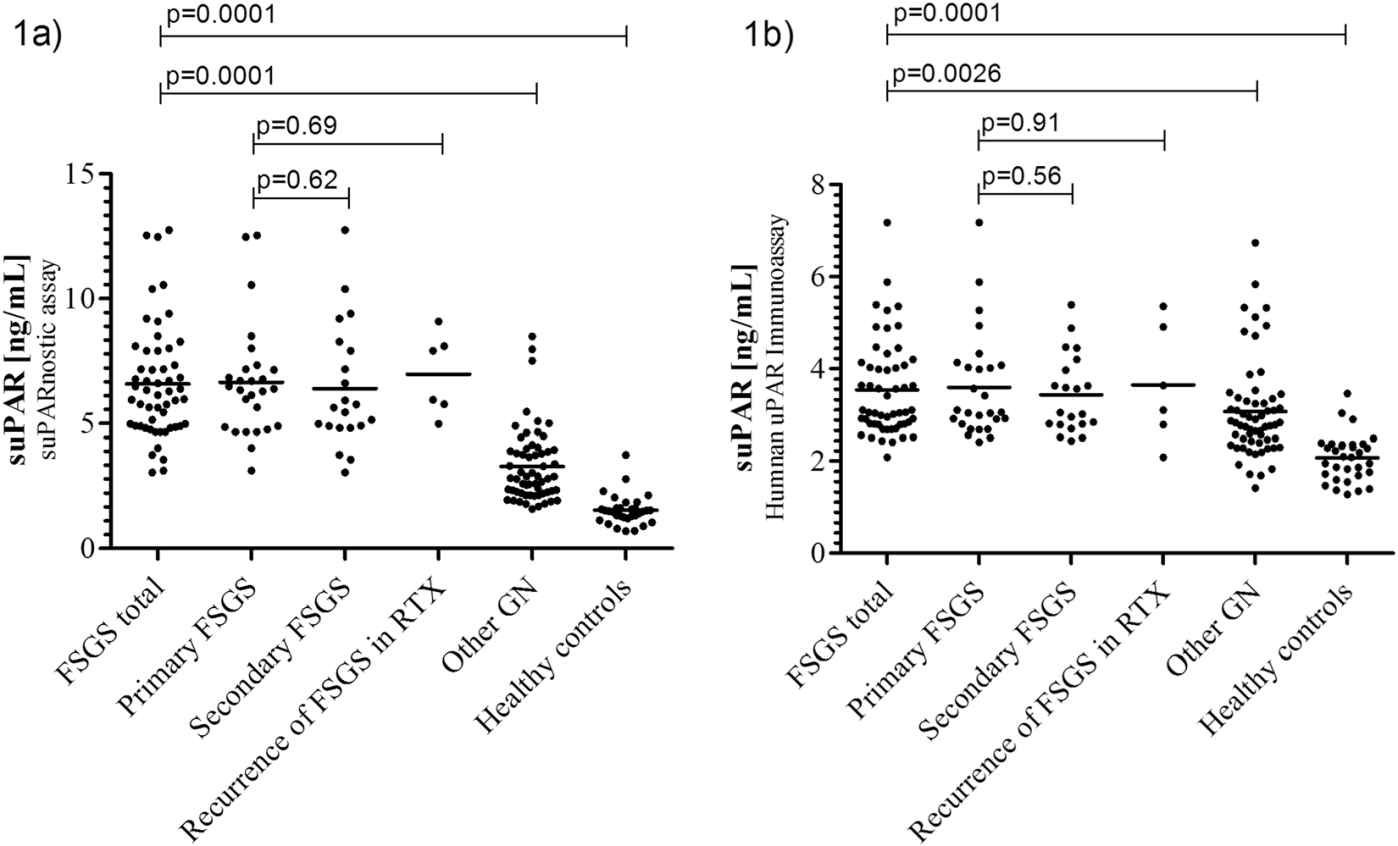
Plasma suPAR levels in patients with FSGS total, primary FSGS, secondary FSGS, recurrence of FSGS after renal transplantation, other glomerulonephritis and healthy controls. (**a**) Results from suPARnostic™ assay: plasma suPAR levels of patients with FSGS (FSGS total, 6.58 ± 2.25 ng/mL) were significantly higher than in patients with other GN (3.28 ± 1.47 ng/mL) and healthy controls (1.53 ± 0.60 ng/mL). (**b**) Results from Quantikine Human uPAR ELISA Kit: suPAR levels in patients with FSGS (FSGS total, 3.54 ± 1.03 ng/mL) were significantly higher than in patients with other GN (3.07 ± 1.06 ng/mL) and healthy controls (2.07 ± 0.50 ng/mL). There was no significant difference in plasma suPAR levels between patients with primary FSGS, secondary FSGS and recurrence of FSGS after renal transplantation. P-values are based on the Mann-Whitney U test. Abbreviations: FSGS, focal segmental glomerulosclerosis; GN, glomerulonephritis; RTX, renal transplantation.

Patients with FSGS presented in general higher suPAR levels [ng/mL] compared to other GN patients and healthy individuals (6.58 ± 2.25, vs. 3.28 ± 1.47 vs. 1.53 ± 0.60 ng/mL; p = 0.0001) as measured with the suPARnostic™ assay (Table 1, Fig. 1a). Also when using the Quantikine Human uPAR ELISA Kit significantly higher suPAR levels [ng/mL] were obtained for FSGS patients compared to patients with other GN and healthy volunteers (3.54 ± 1.03, vs. 3.07 ± 1.06 vs. 2.07 ± 0.50 ng/mL; p = 0.0026), (Table 1, Fig. 1b). No difference was found between plasma suPAR levels of patients with primary FSGS, secondary FSGS or recurrence of FSGS post transplantation. A comprehensive subgrouping of patients with FSGS, other GN and healthy individuals is depicted in Supplementary Table S1.

In a group-specific comparison of the suPARnostic™ assay and the Quantikine Human uPAR-Immunoassay the mean difference of suPAR levels in FSGS patients was 3.04 ± 1.5 ng/mL (44%; 95% CI, 40 to 48; p < 0.001), in patients with other GN 0.21 ± 1.1 ng/mL (−2%; 95% CI, −11 to 6; p < 0.62) and in healthy controls −0.54 ± 0.3 ng/mL (−43%; 95% CI, −54 to −32; p < 0.001).

### Plasma suPAR level depending on kidney function

The mean eGFR was significantly lower in FSGS patients compared to patients with other GN or healthy volunteers, which might partially account for the difference of suPAR levels between groups. Multiple regression analysis showed that baseline suPAR level was significantly correlated with eGFR (p = 0.001) which remained significant after multivariable adjustment as shown in Table 2 for both ELISA tests. No significant association was observed between plasma suPAR and gender, age, proteinuria, presence of microscopic hematuria, history of renal transplantation or duration of FSGS disease.

**Table 2:** Association of baseline variables with plasma suPAR levels, measured by two independent ELISA tests, in FSGS patients.

Variable	Regression coefficient (95% CI) with suPAR adjusted^a^ suPARnostic™ assay*	P-value	Regression coefficient (95% CI) with suPAR adjusted^a^ Quantikine Human uPAR ELISA Kit**	P-value
Gender (male)	−0.598 (−1.748 to 0.553)	0.302	−0.341 (−0.876 to 0.193)	0.205
Age (years)	0.009 (−0.028 to 0.046)	0.641	0.015 (−0.002 to 0.032)	0.089
eGFR (mL/min/1.73 m^2^)	−0.043 (−0.061 to −0.025)	0.001	−0.018 (−0.026 to −0.010)	0.001
Serum creatinine (mg/dL)	0.074 (−0.576 to 0.428)	0.769	−0.001 (−0.234 to 0.233)	0.996
Serum albumin (g/dL)	0.003 (−0.099 to 0.105)	0.952	−0.002 (−0.049 to 0.046)	0.941
Serum C-reactive protein (mg/dL)	0.492 (−0.661 to 1.645)	0.395	0.101 (−0.438 to 0.640)	0.709
Serum total cholesterol (mg/dL)	−0.004 (−0.014 to 0.006)	0.435	−0.001 (−0.005 to 0.004)	0.757
Urine protein/creatinine ratio (mg/g)	0.001 (−0.001 to 0.001)	0.142	0.001 (−0.001 to 0.001)	0.100
Urine albumin/creatinine ratio (mg/g)	0.001 (−0.001 to 0.002)	0.871	−0.001 (−0.001 to 0.002)	0.942
Presence of microscopic hematuria	−0.466 (−2.222 to 1.290)	0.595	−0.133 (−0.927 to 0.661)	0.738
Presence of hypertension	−1.843 (−4.227 to 0.541)	0.125	−0.026 (−1.130 to 1.077)	0.961
Presence of diabetes	0.855 (−0.726 to 2.437)	0.280	0.476 (−0.200 to 1.151)	0.162
History of renal transplantation	0.592 (−0.611 to 1.795)	0.327	0.111 (−0.453 to 0.674)	0.695
Duration of disease (months)	0.023 (−0.099 to 0.146)	0.702	0.026 (−0.031 to 0.082)	0.367

CI, confidence interval; eGFR, estimated glomerular filtration rate.

^a^Multivariable adjustment for sex (male/female), age (years), eGFR and urine protein/creatinine ratio.

*Plasma suPAR values were measured by suPARnostic™ assay.

**Plasma suPAR values were measured by Quantikine Human uPAR ELISA Kit.

To test the validity of suPAR levels in patients without chronic kidney disease, we excluded 54 patients with eGFR below 60 mL/min, in accordance with the definition of chronic kidney disease, for a subanalysis. In the remaining 92 study subjects with eGFR ≥60 mL/min a significant difference in suPAR levels [ng/mL] between FSGS and other GN patients (5.05 ± 1.35 vs. 2.91 ± 0.94 ng/mL, p = 0.001 for the suPARnostic™ assay and 2.91 ± 0.48 vs. 2.61 ± 0.49 ng/mL, p = 0.036 for the Quantikine Human uPAR ELISA Kit) was confirmed. This also applied to the comparison between FSGS patients and healthy controls (5.05 ± 1.35 vs. 1.53 ± 0.60 ng/mL, p = 0.001 for the suPARnostic™ assay and 2.91 ± 0.48 vs. 2.07 ± 0.50 ng/mL, p = 0.001 for the Quantikine Human uPAR ELISA Kit). Of note, renal function parameters did not differ between FSGS, other GN patients and healthy controls in this subanalysis (eGFR 90.2 ± 19.1. vs. 93.5 ± 20.0 vs. 99.9 ± 15.9 mL/min; FSGS vs. other GN: p = 0.60, FSGS vs. healthy controls: p = 0.12; Table 3). Characteristics and laboratory parameters of FSGS patients with eGFR above 60 mL/min and below are presented in Supplementary Table S2.

**Table 3:** Plasma suPAR levels in patients with FSGS, other GN and healthy controls with glomerular filtration rate ≥ 60 mL/min.

	FSGS total	Other GN	Healthy controls	P-value FSGS vs. Other GN	P-value FSGS vs. Healthy
Number of subjects	18	42	32		
Age (years)	45.2 ± 14.6	46.2 ± 15.6	42.3 ± 8.3	0.82	0.18
Gender (male/female)	7/11	28/15	16/16	0.06	0.58
Serum creatinine (mg/dL)	0.87 ± 0.18	0.90 ± 0.19	0.86 ± 0.11	0.51	0.98
eGFR (mL/min/1.73 m^2^)	90.2 ± 19.1	93.5 ± 20.0	99.9 ± 15.9	0.60	0.12
Plasma suPAR (ng/mL)*	5.05 ± 1.35	2.91 ± 0.94	1.53 ± 0.60	0.001	0.001
Plasma suPAR (ng/mL)**	2.91 ± 0.48	2.61 ± 0.49	2.07 ± 0.50	0.036	0.001

Abbreviations: FSGS, focal segmental glomerulosclerosis; GN, glomerulonephritis.

Plus-minus values indicate means ± standard deviation.

*Plasma suPAR value measured by suPARnostic™ assay.

**Plasma suPAR value measured by Quantikine Human uPAR ELISA Kit.

### Sensitivity and specificity of plasma suPAR for FSGS diagnosis

We performed a receiver operating characteristic (ROC) analysis to determine whether suPAR is a potent marker to diagnose and differentiate FSGS.

**Figure 2:**
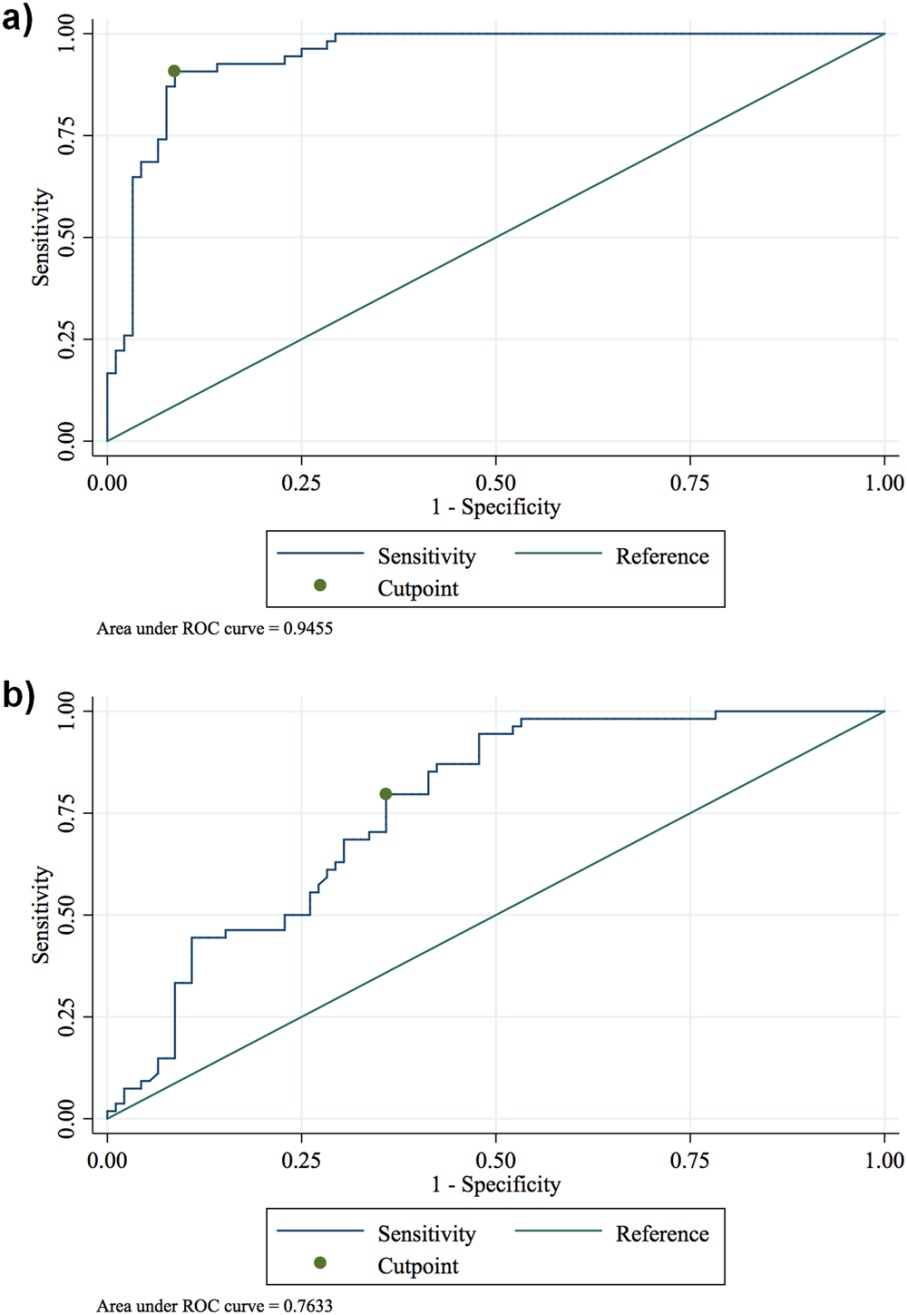
Receiver operating characteristic (ROC) analysis of the plasma soluble urokinase-type plasminogen activator receptor (suPAR) in patients FSGS vs. non-FSGS. (**a**) For the cut-off value of 4.644 ng/mL measured by the suPARnostic™ assay, sensitivity and specificity were both 0.91 with an AUC of 0.946 [95% CI 0.91 to 0.98]. (**b**) For the cut-off value of 2.789 ng/mL measured by the Quantikine Human uPAR ELISA Kit the sensitivity and specificity were 0.80 and 0.64, respectively with an AUC of 0.763 [95% CI 0.69 to 0.83].

By identifying the point at which the difference between sensitivity and 1-specificity is maximal, we determined that the optimal cut-off value is 4.644 ng/mL of the suPAR concentration measured by the suPARnostic™ assay for FSGS vs. non-FSGS patients [95% CI 4.02 to 5.27]. For this cut-off value, the sensitivity and specificity of the test were both 0.91 with an AUC of 0.946 [95% CI 0.91 to 0.98] (Fig. 2a).

Using the Quantikine Human uPAR ELISA Kit the optimal cut-off is 2.789 ng/mL for FSGS vs. non-FSGS patients [95% CI 2.35 to 3.22]. For this cut-off value, the sensitivity and specificity of the test were 0.80 and 0.64, respectively with an AUC of 0.763 [95% CI 0.69 to 0.83] (Fig. 2b).

These results suggest that both kits measuring suPAR can be used to distinguish FSGS from non-FSGS, with the suPARnostic™ assay performing a higher differentiation in our analysis.

An illustration of suPAR cut-off values and possible false negative test results in patients with FSGS depending on the test method used is shown in Supplementary Fig. S2.

The ROC analysis of a subanalysis of subjects with eGFR ≥60 mL/min is presented in Supplementary Fig. S3.

### Prognostic assessment of plasma suPAR levels

A multivariate analysis was developed including age, sex, eGFR and urine protein/creatinine ratio to test the prognostic value of plasma suPAR in FSGS patients. With each suPAR increase by one point [ng/mL], measured with the suPARnostic™ assay following changes were detected: decrease in the mean annual delta eGFR by 0.098 mL/min (95% CI −0.66 to 0.46), p = 0.729; an increase in the mean annual delta urine protein/creatinine ratio by 48.3 mg/g (95% CI −44.5 to 141), p = 0.30 as well as in the mean annual delta urine albumin/creatinine ratio by 9.2 mg/g (95% CI −93.5 to 111.9), p = 0.86. These results were not significant. During the 5-year follow-up, 12 out of 54 patients with FSGS developed ESRD and 4 FSGS patients died. With each suPAR increase by one point [ng/mL] the odds ratio for death increased by 1.15 ± 0.24 (95% CI 0.76 to 1.72), p = 0.52. A significant association could be shown between suPAR and progression to ESRD, as with each suPAR increase by one point [ng/mL] the odds ratio for ESRD increased by 1.34 ± 0.19 (95% CI 1.01 to 1.77), p = 0.046. Corresponding results obtained with the Quantikine Human uPAR ELISA Kit are also given in Supplementary Table S3. A graphical depiction of increasing plasma suPAR levels and progression to ESRD is presented in Supplementary Fig. S4.

Similar results were obtained in a model used as sensitivity analysis in which differences in eGFR, urine albumin/creatinine ratio and protein/creatinine ratio values were set to zero after loss of renal function compared to the original model using missing data. **(**Supplementary Table S3).

## Discussion

This study was conducted to test the diagnostic and prognostic value of suPAR as a biomarker for FSGS. Since conflicting results about the clinical role of suPAR in the literature are derived from different ELISA methods measuring suPAR plasma levels, two of the leading assays were used in a side-by-side comparison and their results were compared in this study.

Our study analyzed plasma suPAR levels in patients with different forms of FSGS (primary and secondary FSGS as well as recurrence after transplantation), other types of glomerulonephritis and healthy subjects. Furthermore, suPAR was assessed according to estimated glomerular filtration rate in patients with and without chronic kidney disease. The prognostic evaluation was performed over a period of five years with respect to renal retention parameters, proteinuria, disease progression and mortality. Our data show that FSGS patients have significantly higher suPAR plasma levels when compared to patients with other forms of GN or healthy controls indicating the significance of suPAR tests in FSGS diagnosis. Furthermore, elevated suPAR values in FSGS patients were found to have a predictive value for disease progression to ESRD. Our results also demonstrate that both commercially available suPARnostic™ assay and the Quantikine Human uPAR Immunoassay can be used to differentiate FSGS from non-FSGS based on suPAR plasma levels, with the suPARnostic™ assay being more FSGS specific. Novel test specific cut-offs of 4.644 ng/mL for the suPARnostic™ assay and 2.789 ng/mL for the Quantikine Human uPAR Immunoassay were established for FSGS diagnosis in the entire study population.

A number of FSGS studies have used different test methods for the quantitative measurement of suPAR and came to different conclusions. It is noteworthy that fragments of suPAR are not recognized equally by the currently available suPAR ELISA test methods. The separation of uPAR from its GP anchor releases suPAR and further suPAR fragments are generated by additional cleavage between the D1, D2/D3 domains^17^. Furthermore, depending on the amount of glycosylation, the size of the cleaved suPAR fragments ranges from 25 to 50 kDa^17^. This is of central importance: suPAR fragments differ in regard to their biological relevance and are not equally detected in different suPAR assays leading to varying results which are hard to compare among studies^5,38,39^. In our study two ELISA tests were used, the suPARnostic™ assay and the Quantikine Human uPAR immunoassay. While the suPARnostic™ assay represents a double monoclonal antibody sandwich assay, the Quantikine Human uPAR immunoassay uses a monoclonal capture antibody and a polyclonal detection antibody. Furthermore, the suPARnostic™ assay measures both full and cleaved forms of suPAR, which might explain variations in test readings. Thus, although both ELISA kits tested in our study were capable to differentiate FSGS from non-FSGS, test results were not completely identical: in the entire study population of 146 subjects the suPARnostic™ assay outperfomed the Quantikine Human uPAR-Immunoassay in differentiating FSGS from non-FSGS. This was based on higher suPAR levels in patients with FSGS and lower suPAR levels in healthy volunteers when compared with the Quantikine Human uPAR-Immunoassay. This difference between the two test kits used was attenuated in subjects with eGFR above 60 mL/min.

Our study demonstrates the importance of suPAR testing in the diagnosis of FSGS. In previous studies, the correlation of suPAR with renal insufficiency was repeatedly discussed critically^40^. This led us to analyze suPAR values in patients with chronic kidney disease (according to the chronic kidney disease (CKD) definition of GFR below 60 mL/min) as well as in patients without CKD. The inverse association of suPAR with eGFR was confirmed in our study; moreover the significance of suPAR in FSGS assessment independent of renal function could be emphasized.

SuPAR may not only be a diagnostic biomarker for FSGS, but also a pathogenic factor for progression of disease^26^. Previously, it has been shown that proteinuria can be reduced by eliminating suPAR from the circulation using extracorporeal methods, e.g. plasmapheresis and immunoadsorption. Recently, also hemoadsorption by means of polymeric adsorber columns have been used to remove suPAR^38^. As suPAR has a high affinity to protein A immunoapheresis columns, it is removable from the circulation, at least in part, but also rebounds within days^16^. An established therapy for recurrence of FSGS after transplantation is plasmapheresis or immunoadsorption, in combination with steroids, cyclosporine A and rituximab or alone^15,38^.

Recurrence of FSGS after transplantation manifests in about 30% of FSGS patients receiving their first kidney transplant^2^ and in up to 80% of patients after second transplantation with a history of transplant loss due to recurrence of FSGS in the first graft^41^. This can even manifest within hours post transplantation^42^. Since the recurrence of disease is a serious problem, there are attempts to reduce recurrence rates by using induction therapies such as rituximab prior to transplantation in patients with FSGS as underlying disease with high suPAR values^43^. Accordingly, suPAR values in kidney transplant candidates where a high recurrence rate can be assumed could serve as guide to a patient-tailored immunosuppressive therapy, but randomized controlled trials are needed to support this hypothesis.

Furthermore, suPAR measurement could be particularly useful in other clinical situations, where a kidney biopsy for diagnosis is not possible. With the aid of other established biomarkers such as anti-PLA2R antibodies^44^, different forms of GN (membranous nephropathy versus FSGS) can be distinguished, which has in turn therapeutic consequences. Thus, suPAR is of manifold interest in FSGS pathogenesis, diagnosis, therapy and prognosis.

The main limitation of this study is its retrospective design. In addition, our sample size was relatively small due to the monocentric nature of the study. Furthermore, no urine samples were taken that would have allowed further potentially significant analyses^34,45^. Our study was strengthened by a well-characterized cohort of patients with primary FSGS, secondary FSGS, recurrence of FSGS after transplantation, different forms of glomerulonephritis and healthy controls with long follow-up resulting in clear results: plasma suPAR levels do not distinguish between patients with primary and secondary form of FSGS and recurrence of disease after renal transplantation, which is in line with results from previous studies^46^. But suPAR levels are useful to differentiate FSGS patients from patients with other forms of glomerulonephritis and healthy controls, e.g. when renal biopsies are difficult. The independence of suPAR from eGFR has been demonstrated and both the suPARnostic™ assay and the Quantikine Human uPAR-Immunoassay are capable for FSGS identification. Furthermore, the prognostic value of suPAR for the progression to end-stage renal disease was identified.

The scope of this study was not to establish the role of suPAR in the pathogenesis of FSGS. The main aim was to examine the significance of suPAR in FSGS diagnosis and different courses of disease. Hereby, we describe new cut-offs for suPAR using two different suPAR ELISA assays. The comparison of these two assays, showing relevant differences, may explain varying results obtained in previous studies using different detection assays. Therefore, suPAR values have to be interpreted in the context of population and the test method used. Knowledge of test specific cut-offs makes suPAR a valuable biomarker for FSGS.

## Supplementary information

FSGS Supplementary Information

## Data Availability

The datasets generated in the context of this paper are made available by the corresponding author on request.
