# Supplementary material for: Diagnostic and Prognostic Value of Soluble Urokinase-type Plasminogen Activator Receptor (suPAR) in Focal Segmental Glomerulosclerosis and Impact of Detection Method
Source: Sci Rep. 2019 Sep 24;9:13783. doi: 10.1038/s41598-019-50405-8 (PMC6760112; doi:10.1038/s41598-019-50405-8)
Supplement: Supplementary file 1 — FSGS Supplementary Information [file 41598_2019_50405_MOESM1_ESM.doc]

**Diagnostic and Prognostic Value of Soluble Urokinase-type Plasminogen Activator Receptor (suPAR) in Focal Segmental Glomerulosclerosis and Impact of Detection Method**

Wolfgang Winnicki^1^, Gere Sunder-Plassmann^1^, Gürkan Sengölge^1^, Ammon Handisurya^1^, Harald Herkner^2^, Christoph Kornauth^3^, Bernhard Bielesz^1*^, Ludwig Wagner^1^, Željko Kikić^1^, Sahra Pajenda^1^, Thomas Reiter^1^, Benjamin Schairer^1^, Alice Schmidt^1^

^1^Department of Medicine III, Division of Nephrology and Dialysis, Medical University of Vienna, Austria

^2^Department of Emergency Medicine, Medical University of Vienna, Austria

^3^Clinical Institute of Pathology, Medical University of Vienna, Vienna, Austria

^*^ Corresponding author

E-mail: bernhard.bielesz@meduniwien.ac.at

**Supplementary Figure S1. Schematic illustration of the suPARnostic™ assay and the Quantikine Human uPAR ELISA Kit.**

**
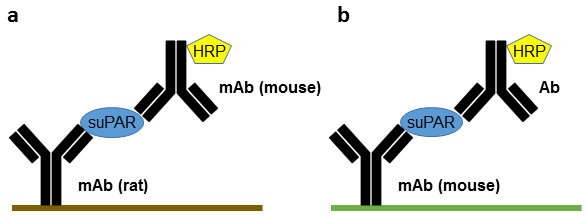
**

Abbreviations: Ab, polyclonal antibody; HRP, horseradish peroxidase; mAb, monoclonal antibody, suPAR, soluble urokinase-type plasminogen activator receptor.

a) The suPARnostic® ELISA (Virogates) is a classical double monoclonal antibody sandwich ELISA with direct labeling of the detection antibody. Two monoclonal antibodies, a rat anti-human suPAR antibody as capture antibody and a HRP-conjugated mouse anti-human suPAR as detecting antibody are used.

b) The Quantikine Human uPAR Immunoassay (R&D Systems) is a sandwich ELISA as well. The capturing antibody is a mouse monoclonal antibody, while a HRP-conjugated polyclonal antibody is used for detection.

**Supplementary Figure S2. SuPAR cut-off values and possible false negative test results in patients with FSGS depending on the test method used (Kit A: suPARnostic™ assay; Kit B: Quantikine Human uPAR ELISA Kit).**


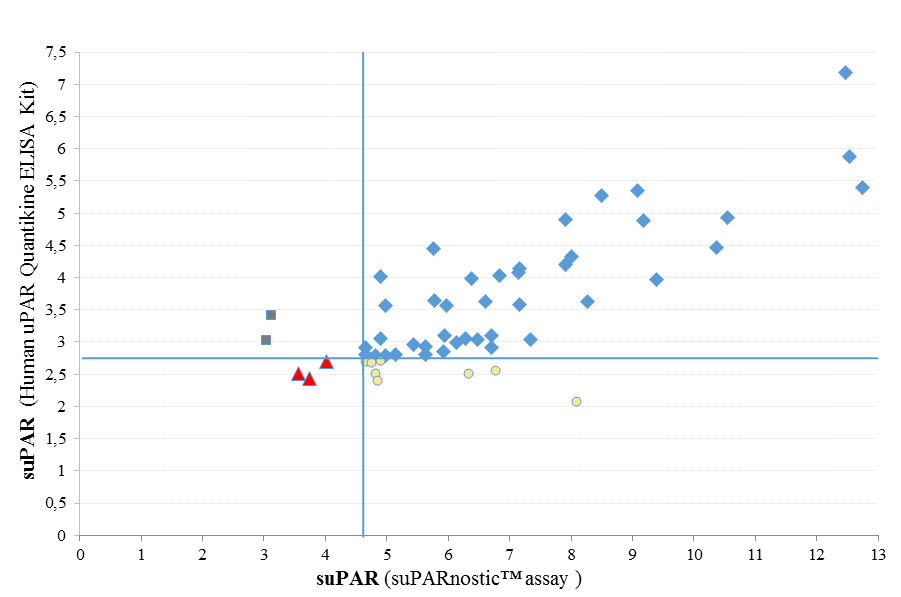


Patients with positive results detected in both kit A and kit B are marked with a rhombus (◊). Patients with a positive result detected in kit A, but negative result in kit B are marked with a circle (○). Patients with a positive result detected in kit B, but negative result in kit A are marked with a quad (□). Patients with negative results detected in both kit A and kit B are marked with a triangle (▲).

**Supplementary Figure S3. Receiver operating characteristic (ROC) analysis of the plasma soluble urokinase-type plasminogen activator receptor (suPAR) in FSGS vs. non-FSGS patients with** **eGFR ≥ 60 mL/min.**


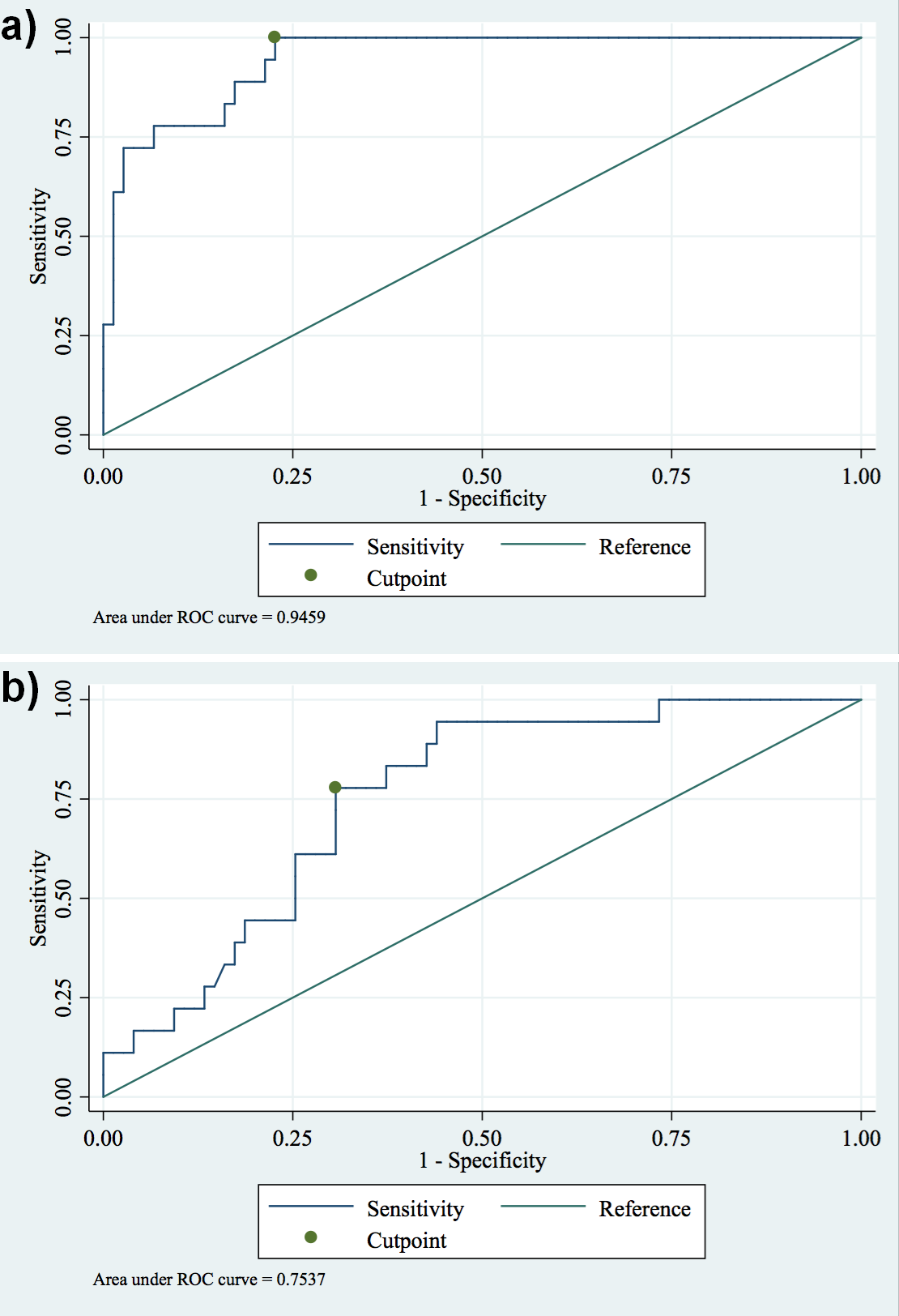


a) For the cut-off value of 3.041 ng/mL measured by the suPARnostic™ assay, sensitivity and specificity were 1.00 and 0.77, respectively with an AUC of 0.946 [95% CI 0.90 to 0.99].

b) For the cut-off value of 2.671 ng/mL measured by the Quantikine Human uPAR ELISA Kit, the sensitivity and specificity were 0.78 and 0.69, respectively with an AUC of 0.754 [95% CI 0.65 to 0.86].

**Supplementary Figure S4.** **Plasma suPAR and progression to end-stage renal disease.**


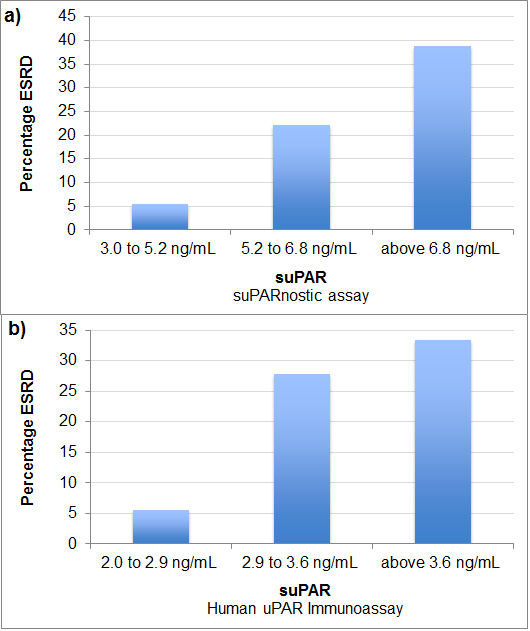


Abbreviations: ESRD, end-stage renal disease, suPAR, soluble urokinase-type plasminogen activator receptor.

Higher plasma suPAR values are predictive for progression to end-stage renal disease in FSGS patients (n=12). Each bar represents terziles corresponding to 4 patients per group.

**Supplementary Table S1. Subgroup analysis with detailed segmentation of patients with FSGS, other GN and healthy individuals**.

|  | **suPAR*** | **P-value** | **suPAR**** | **P-value** |
| --- | --- | --- | --- | --- |
| **Primary FSGS (n=27)** | 6.65 ± 2.26 |  | 3.60 ± 1.14 |  |
| **Secondary FSGS (n=21)** | 6.40 ± 2.46 | 0.615 | 3.44 ± 0.85 | 0.563 |
| **Recurrence of FSGS in RTX (n=6)** | 6.96 ± 1.61 | 0.687 | 3.65 ± 1.26 | 0.911 |
| **Membranous GN (n=38)** | 3.30 ± 1.48 | 0.001 | 3.27 ± 1.05 | 0.172 |
| **Minimal Change GN (n=6)** | 3.08 ± 1.07 | 0.001 | 2.41 ± 0.37 | 0.006 |
| **Immune Complex GN (n=6)** | 3.50 ± 2.55 | 0.001 | 3.48 ± 1.49 | 0.789 |
| **Anti-GBM GN (n=2)** | 2.91 ± 0.17 | 0.004 | 2.38 ± 1.36 | 0.082 |
| **IgA GN (n=1)** | 2.14 | 0.011 | 3.12 | 0.619 |
| **Lupus GN (n=1)** | 1.86 | 0.007 | 2.87 | 0.451 |
| **Interstitial Nephritis (n=1)** | 4.90 | 0.322 | 2.77 | 0.389 |
| **Unspecified GN (n=5)** | 3.50 ± 0.66 | 0.001 | 2.20 ± 0.63 | 0.003 |
| **Healthy Individuals (n=32)** | 1.53 ± 0.60 | 0.001 | 2.07 ± 0.50 | 0.001 |

Abbreviations: GN, glomerulonephritis; FSGS, focal segmental glomerulosclerosis; RTX, renal transplantation.

Plus-minus values are means ± standard deviation.

* plasma suPAR value (ng/mL) measured by suPARnostic™ assay

** plasma suPAR value (ng/mL) measured by Quantikine Human uPAR ELISA Kit

No difference between plasma suPAR levels of patients with primary FSGS, secondary FSGS or recurrence of FSGS after transplantation is shown. However, plasma suPAR levels of patients with primary FSGS were significantly higher than in patients with other subtypes of glomerulonephritis and healthy volunteers, irrespective of the assay used.

**Supplementary Table S2. Plasma suPAR levels in patients with FSGS and glomerular filtration rate above and below** **60mL/min.**

|  | **Entire FSGS cohort** | **FSGS cohort with eGFR ≥ 60 mL/min** | **FSGS cohort with eGFR < 60 mL/min** | **P-value** |
| --- | --- | --- | --- | --- |
| Number of subjects | 54 | 18 | 36 |  |
| **Characteristics** |  |  |  |  |
| - Age (years) | 45.1 ± 14.7 | 45.2 ± 14.6 | 45.0 ± 14.7 | 0.98 |
| - White - number (%) | 48 (89) | 16 (89) | 32 (89) | 0.57 |
| **Laboratory parameters** |  |  |  |  |
| - Serum albumin (g/dL) | 37.47 ± 7.27 | 37.33 ± 6.67 | 37.55 ± 7.46 | 0.74 |
| - Serum total cholesterol (mg/dL) | 218.40 ± 62.08 | 232.78 ± 58.49 | 210.31 ± 61.60 | 0.14 |
| - Serum C-reactive protein (mg/dL) | 0.41 ± 0.47 | 0.51 ± 0.65 | 0.36 ± 0.35 | 0.39 |
| - Serum creatinine (mg/dL) | 2.19 ± 1.89 | 0.87 ± 0.18 | 2.85 ± 1.98 | <0.0001 |
| - eGFR (mL/min/1.73m^2^) | 52.37 ± 31.61 | 90.22 ± 19.07 | 33.44 ± 14.64 | <0.0001 |
| - Microscopic hematuria, n (%) | 8 (15) | 1 (6) | 7 (19) | 0.17 |
| - Urine albumin/creatinine ratio (mg/g) | 1582 ± 1728 | 1835 ± 2077 | 1472 ± 1507 | 0.99 |
| - Urine protein/creatinine ratio (mg/g) | 2316 ± 2325 | 2663 ± 2599 | 2142 ± 2119 | 0.77 |
| **Plasma suPAR levels (ng/mL)** |  |  |  |  |
| SuPAR (suPARnostic™ assay)* | 6.58 ± 2.25 | 5.05 ± 1.35 | 7.35 ± 2.19 | 0.0001 |
| SuPAR (Quantikine Human uPAR) ****** | 3.54 ± 1.03 | 2.91 ± 0.48 | 3.86 ± 1.08 | 0.0005 |

Abbreviations: eGFR, estimated glomerular filtration rate; FSGS, focal segmental glomerulosclerosis.

Plus-minus values are means ± standard deviation. Numbers in brackets indicate percentage

* plasma suPAR value (ng/mL) measured by suPARnostic™ assay

** plasma suPAR value (ng/mL) measured by Quantikine Human uPAR ELISA Kit

Patients with FSGS and eGFR above and below 60 mL/min were differentiated and analyzed for plasma suPAR levels. Baseline characteristics and laboratory parameters (except renal function parameters) did not differ between groups.

**Supplementary Table S3. Prognostic assessment of plasma suPAR levels and linear mixed models to assess inter-group differences regarding missing data.**

| **Linear mixed model** | **Change per point**  **suPAR* raise (95% CI)** | **P-value** | **Change per point**  **suPAR** raise (95% CI)** | **P-value** |
| --- | --- | --- | --- | --- |
| **Model I** |  |  |  |  |
| eGFR slope | -0.098 (-0.659 to 0.4645) | 0.729 | 0.5805 (-0.651 to 1.812) | 0.348 |
| Urine p/c ratio | 48.32 (-93.54 to 111.96) | 0.30 | 3.45 (-208.44 to 215.35) | 0.974 |
| Urine a/c ratio | 9.209 (-93.54 to 111.96) | 0.86 | -59.57 (-293.93 to 174.78) | 0.61 |
| **Model II** |  |  |  |  |
| eGFR slope | 0.0119 (-0.494 to 0.5177) | 0.963 | 0.7434 (-0.3496 to 1.8364) | 0.178 |
| Urine p/c ratio | 28.95 (-19.63 to 77.52) | 0.237 | 10.44 (-96.79 to 117.68) | 0.846 |
| Urine a/c ratio | 9.52 (-21.57 to 40.62) | 0.542 | 11.71 (-56.20 to 79.63) | 0.731 |

Abbreviations: CI, confidence interval; eGFR, estimated glomerular filtration rate; urine a/c ratio, urine albumin/creatinine ratio; urine p/c ratio, urine protein/creatinine ratio.

A multivariate analysis including sex, age, serum creatinine, albumin and CRP was performed.

In model I differences in eGFR, urine protein/creatinine ratio and urine albumin/creatinine ratio values after loss of renal function was counted as missing.

In model II (sensitivity analysis) differences in eGFR, urine protein/creatinine ratio and urine albumin/creatinine ratio values after loss of renal function were set to zero.

* plasma suPAR value (ng/mL) measured by suPARnostic™ assay

** plasma suPAR value (ng/mL) measured by Quantikine Human uPAR ELISA Kit

The sensitivity analysis in which differences in eGFR, urine albumin/creatinine ratio and protein/creatinine ratio values were set to zero after loss of renal function - compared to the original model using missing data - revealed similar results.
